# Multilevel allometric modelling of maximal stroke volume and peak oxygen uptake in 11–13-year-olds

**DOI:** 10.1007/s00421-019-04241-3

**Published:** 2019-10-17

**Authors:** Neil Armstrong, Jo Welsman

**Affiliations:** Children’s Health and Exercise Research Centre, University of Exeter, St Lukes Campus, Heavitree Road, Exeter, EX1 2LU UK

**Keywords:** Adolescents, Aerobic fitness, Body composition, Cardiovascular variables, Cardiorespiratory fitness, Children

## Abstract

**Purpose:**

To investigate (1) whether maximal stroke volume (SV_max_) occurs at submaximal exercise intensities, (2) sex differences in SV_max_ once fat-free mass (FFM) has been controlled for, and, (3) the contribution of concurrent changes in FFM and SV_max_ to the sex-specific development of peak oxygen uptake $\left( {{\dot{\text{V}}\text{O}}_{2} } \right)$.

**Methods:**

The peak ${\dot{\text{V}}\text{O}}_{2}$ s of 61 (34 boys) 11–12-year-olds were determined and their SV determined during treadmill running at 2.28 and 2.50 m s^−1^ using carbon dioxide rebreathing. The SV_max_ and peak ${\dot{\text{V}}\text{O}}_{2}$ of 51 (32 boys) students who volunteered to be tested treadmill running at 2.50 m s^−1^ on three annual occasions were investigated using multilevel allometric modelling. The models were founded on 111 (71 from boys) determinations of SV_max_, FFM, and peak ${\dot{\text{V}}\text{O}}_{2}$.

**Results:**

Progressive increases in treadmill running speed resulted in significant (*p* < 0.01) increases in ${\dot{\text{V}}\text{O}}_{2}$, but SV levelled-off with nonsignificant (*p* > 0.05) changes within ~ 2–3%. In the multilevel models, SVmax increased proportionally to FFM^0.72^ and with FFM controlled for, there were no significant (*p* > 0.05) sex differences. Peak ${\dot{\text{V}}\text{O}}_{2}$ increased with FFM but after adjusting for FFM^0.98^, a significant (*p* < 0.05) sex difference in peak ${\dot{\text{V}}\text{O}}_{2}$ remained. Introducing SV_max_ to the multilevel model revealed a significant (*p* < 0.05), but small additional effect of SVmax on peak ${\dot{\text{V}}\text{O}}_{2}$.

**Conclusions:**

Fat-free mass explained sex differences in SV_max_, but with FFM controlled for, there was still a ~ 5% sex difference in peak ${\dot{\text{V}}\text{O}}_{2}$. SV_max_ made a modest additional contribution to explain the development of peak ${\dot{\text{V}}\text{O}}_{2} ,$ but there remained an unresolved sex difference of ~ 4%.

## Introduction

Peak oxygen uptake (${\dot{\text{V}}\text{O}}_{2}$), the highest ${\dot{\text{V}}\text{O}}_{2}$ elicited during an incremental exercise test to exhaustion, is internationally recognized as the criterion measure of youth cardiorespiratory fitness. Laboratory-based studies of boys’ peak ${\dot{\text{V}}\text{O}}_{2}$ date back over 80 years (Robinson 1938) and data on both sexes have been available for over 65 years (Åstrand 1952), but the role of concurrent changes in morphological and physiological variables in the development of peak ${\dot{\text{V}}\text{O}}_{2}$ during growth and maturation remains to be fully elucidated (Armstrong and McManus 2017).

The extant literature consists largely of cross-sectional studies and the interpretation of data has been clouded by inappropriate ratio scaling of peak ${\dot{\text{V}}\text{O}}_{2}$ with body mass (Welsman and Armstrong 2019). Allometric scaling of peak ${\dot{\text{V}}\text{O}}_{2}$ data has enhanced understanding of youth cardiorespiratory fitness (Armstrong et al. 1998; Loftin et al. 2016; Welsman et al. 1996), but cross-sectional studies only provide a ‘snapshot’ of a continuous process. There are few rigorously analysed longitudinal studies, but the emergence (Aitkin et al. 1981), development (Rasbash et al. 2018), and application (Nevill et al. 1998) of multilevel allometric modelling to paediatric exercise physiology has provided new insights into the development of cardiorespiratory fitness. Using multilevel allometric modelling, the effects of age, maturity status, body size, body composition, and physiological covariates can be partitioned concurrently within an allometric framework to provide a sensitive interpretation of the development of peak ${\dot{\text{V}}\text{O}}_{2}$. To our knowledge, no appropriate analysis including both morphological and physiological variables has been published.

Recent multilevel allometric models of large datasets have demonstrated the influence of age- and maturity status-driven concurrent changes in body mass and fat-free mass (FFM) on the development of peak ${\dot{\text{V}}\text{O}}_{2}$ (Armstrong and Welsman 2019a, b). Longitudinal analyses of 1057 treadmill determinations of 10–18-year-olds’ peak ${\dot{\text{V}}\text{O}}_{2}$ showed that in both sexes with age and body mass controlled for, maturity status made a positive contribution to explaining peak ${\dot{\text{V}}\text{O}}_{2}$. When FFM was introduced to the multilevel allometric models, the effects of maturity status became nonsignificant (*p* > 0.05) and the models provided a significantly (*p* < 0.05) better statistical fit to the data. The authors argued that in both sexes, the influence of maturity status on peak ${\dot{\text{V}}\text{O}}_{2}$ was largely evidenced through maturation-driven changes in FFM (Armstrong and Welsman 2019a). These findings were confirmed in multilevel allometric modelling analyses of 320 cycle ergometer-determinations of 11–16-year-olds’ peak ${\dot{\text{V}}\text{O}}_{2}$ (Armstrong and Welsman 2019b). However, regardless of exercise modality and with age, maturity status, and FFM controlled for, an unexplained sex difference remained with boys’ peak ${\dot{\text{V}}\text{O}}_{2}$ significantly (*p* < 0.05) higher than that of girls and the difference increasing with age (Armstrong and Welsman 2019a, b).

In healthy youth, pulmonary ventilation does not limit peak ${\dot{\text{V}}\text{O}}_{2}$ (McManus and Armstrong 2017a) so if the unexplained sex difference in peak ${\dot{\text{V}}\text{O}}_{2}$ lies in its physiological components, cardiovascular variables are the prime candidates. The Fick equation describes ${\dot{\text{V}}\text{O}}_{2}$ as the product of arteriovenous oxygen difference (A-VO_2_ diff) and cardiac output (${\dot{\text{Q}}}$), with ${\dot{\text{Q}}}$ a function of heart rate (HR) and stroke volume (SV). Methods of rigorously determining the HR and peak ${\dot{\text{V}}\text{O}}_{2}$ of children and adolescents are well documented (Falk and Dotan 2019; Jones 1997; McManus and Armstrong 2017b), but understanding of cardiovascular responses to exercise, particularly at peak ${\dot{\text{V}}\text{O}}_{2}$ is limited by ethical and methodological challenges. No direct measurements of youth A-VO_2_ diff, ${\dot{\text{Q}}}$, or SV at peak ${\dot{\text{V}}\text{O}}_{2}$ have been reported and there are, therefore, no ‘gold standard’ reference values as data from indirect methods of estimating A-VO_2_ diff, ${\dot{\text{Q}}}$, and SV can only be compared within methodologies (Rowland 2017). Nevertheless, several methods of estimating children’s ${\dot{\text{Q}}}$ during exercise have been developed and carbon dioxide (CO_2_) rebreathing, Doppler echocardiography, and bioimpedance cardiography have proved to be safe, non-invasive, repeatable, and reliable methods for estimating ${\dot{\text{Q}}}$ and, therefore, A-VO_2_ diff, and SV in paediatric exercise studies (Patterson et al. 1982; Warburton and Bredin 2017; Welsman et al. 2005).

HR and A-VO_2_ diff at peak ${\dot{\text{V}}\text{O}}_{2}$ have been consistently reported to be independent of age, body size, body composition, and sex during childhood and early adolescence (Armstrong and McManus 2017; Obert et al. 2003; Rowland 2017). SV is, therefore, the only cardiovascular factor differentiating peak ${\dot{\text{V}}\text{O}}_{2}$ in girls and boys. During progressive upright exercise, SV initially increases and then plateaus at ~ 50% of peak ${\dot{\text{V}}\text{O}}_{2}$ and remains essentially stable (within ± 5%) until exhaustion. This pattern is one of the most consistently observed responses in cardiac exercise physiology and has been demonstrated in healthy, untrained children and adolescents using a range of methodologies including CO_2_ rebreathing (Cunningham et al. 1994; Turley and Wilmore 1997), Doppler echocardiography (Nottin et al. 2002; Rowland et al. 2000b), and bioimpedance cardiography (McNarry et al. 2011, 2014). Values of exercise SV above ~ 50% of peak ${\dot{\text{V}}\text{O}}_{2}$ have been reported to be highly predictive of maximal SV (SV_max_) (Rowland et al. 1999a), characteristic of individuals as well as group means (Rowland et al. 2000b), and can be assumed to reflect SV_max_ (Rowland, 2005).

Direct comparisons of sex differences in SV_max_ are limited by a paucity of studies, small numbers of participants, data from girls being remarkably sparse, and studies predominantly focusing on pre-pubertal children. Studies consistently report boys’ absolute SV_max_ (i.e. in mL) to be higher than girls’ SV_max_ (Rowland et al. 2000a; Turley and Wilmore 1997; Vinet et al. 2003). In both sexes, SV_max_ increases with age and body size (Miyamura and Honda 1973; Nottin et al. 2002; Turley and Wilmore 1997) and to control for body size, SV is conventionally expressed in 1:1 ratio with body surface area (BSA) as the stroke index. Tanner (1949) unequivocally established that ratio scaling of cardiac data with BSA is fallacious and it has been demonstrated that with SV, the most appropriate scaling procedure is a curvilinear allometric model (Batterham et al. 1999; Rowland 2017). Once it has been allometrically scaled with BSA neither age nor maturity status appears to influence SV (McNarry et al. 2011; Rowland et al.1999b).

Stroke volume is, however, closely matched to metabolic demand and should be considered in relation to the active muscle mass rather than BSA. With the experimental challenges of determining active muscle mass, FFM has emerged as the recommended morphological variable with which to scale SV in paediatric exercise physiology. In cross-sectional studies, SV is best expressed in relation to FFM raised to an empirically derived allometric scaling exponent calculated from the participants in the study (Rowland 2017).

Only two studies have analysed children’s SV_max_ allometrically scaled with FFM, in both cases with 10–12-year-old pre-pubertal or pre-menarcheal children. Vinet et al. (2003) reported the sex difference in SV_max_ relative to FFM^0.79^ to be 4.8%, but not statistically significant (*p* > 0.05) and concluded that sex differences in peak ${\dot{\text{V}}\text{O}}_{2}$ are a reflection of sex differences in body composition and not in cardiac functional capacity. Rowland et al. (2000a) reported the sex difference in SV_max_ relative to FFM^0.84^ to be 5.2%, but not statistically significant (*p* > 0.05). These authors, however, argued that despite not being statistically significant, the observed sex difference in SV_max_ in pre-pubertal children was small, but ‘real’ and suggested that sex differences in SV_max_ account for differences in peak ${\dot{\text{V}}\text{O}}_{2}$ between girls and boys once FFM had been controlled for.

In summary, FFM has been demonstrated to exert a powerful influence on the development of peak ${\dot{\text{V}}\text{O}}_{2}$ but even with FFM controlled for, it is evident that there are still sex differences in peak ${\dot{\text{V}}\text{O}}_{2}$ during childhood and adolescence (Armstrong and Welsman 2019a, b). SVmax appears to be the principal potential cardiovascular contributor to an explanation of sex differences in the development of peak ${\dot{\text{V}}\text{O}}_{2}$. Current data interpretations are in conflict, but cross-sectional studies of pre-pubertal children provide limited insights into the development of SV_max_ and its influence on the development of peak ${\dot{\text{V}}\text{O}}_{2}$. The purposes of the present studies are, therefore, to initially confirm that SV plateaus during progressive treadmill running above moderate exercise intensities and then to use multilevel allometric modelling to investigate in childhood and early adolescence (1) sex differences in SV_max_ once FFM has been controlled for, and, (2) the contribution of concurrent changes in FFM and SV_max_ to the sex-specific development of peak ${\dot{\text{V}}\text{O}}_{2}$.

## Methods

### Participants

Students from local state schools participating in a longitudinal study of the development of cardiorespiratory fitness and short-term power output (Armstrong and Welsman 2019a, c) also volunteered to have their ${\dot{\text{Q}}}$ determined. Some of the initial submaximal ${\dot{\text{Q}}}$ data were published as the project progressed (Armstrong and Welsman 2002), but the present datasets have not previously been brought together for analysis and the contribution of SV_max_ to the development of peak ${\dot{\text{V}}\text{O}}_{2}$ has not been addressed. In Study 1, 61 (34 boys) students completed the exercise tests while treadmill running at 2.22 m s^−1^ and 2.50 m s^−1^ with 35 students (27 boys) completing a third stage of treadmill running at 2.78 m s^−1^. For Study 2, 51 (32 boys) of the students agreed to be tested on three annual occasions while running on a treadmill at 2.50 m s^−1^.

### Experimental procedures

#### Determination of resting variables

Participants were well habituated to the laboratory environment, to the laboratory personnel, and to the experimental procedures. Age was computed from date of birth and date of test. Anthropometric measures were taken as described by the International Biological Programme (Weiner and Lourie 1981) and apparatus was calibrated according to the manufacturers’ instructions. Body mass was assessed using Avery balance scales (Avery, Birmingham, UK), stature was measured using a Holtain stadiometer (Holtain, Crmych, Dyfed, UK), and skinfold thicknesses over the triceps and subscapular regions were measured using Holtain skinfold calipers. Maturity status was visually assessed by the Research Centre nurse using the indices for pubic hair (PH) development described by Tanner (1962). FFM was estimated from skinfolds, body mass, and maturity status using the youth-specific equations developed by Slaughter et al. (1988). Haemoglobin (Hb) concentration was determined as the mean value from duplicate fingertip blood samples which were immediately assayed using a Hemo Cue photometer (Clandon Scientific, Farnborough, UK).

#### Determination of exercise variables

Participants attended the Research Centre on two consecutive mornings to complete the required exercise protocols. Peak ${\dot{\text{V}}\text{O}}_{2}$ was determined on day 1 and at a similar time, the following morning SV and associated cardiovascular variables were determined. All exercise tests were preceded by a standardized warm-up. Peak ${\dot{\text{V}}\text{O}}_{2}$ was determined during a discontinuous, incremental exercise test to voluntary exhaustion on a motorized treadmill (Woodway, Cranlea Medical, Birmingham, UK). HR was monitored using an electrocardiograph (Rigel, Morden, UK) and expired respiratory gases were monitored continuously using an Oxycon Sigma on-line gas-analysis system (Cranlea Medical) which was calibrated prior to each test using gases of verified concentration and an appropriate range of flow rates using a Hans Rudolph calibration syringe (Cranlea Medical). The tests began at a treadmill belt speed of 1.94 m s^−1^ (7 km h^−1^) which was increased by 0.28 m s^−1^ (1 km h^−1^) every 3 min until a speed of 2.78 m s^−1^ (10 km h^−1^) was reached. Subsequently, belt speed was held constant and the gradient was incrementally increased by 2.5% every 3 min until voluntary exhaustion. A 1-min rest period separated the exercise stages. The highest 30 s ${\dot{\text{V}}\text{O}}_{2}$ attained was accepted as a maximal index if clear signs of intense exertion (e.g. hyperpnea, facial flushing unsteady gait, profuse sweating) were demonstrated and supported by a respiratory exchange ratio greater than 1.00 and a HR which was levelling-off over the final stages of the test at a value within 5% of the mean maximal HRs (i.e. 202 beats min^−1^) we have previously reported for large groups of similar-aged young people using the same test protocol (Armstrong et al. 1991). All participants reported in this study satisfied these criteria.

On the following morning, using the same apparatus including the ${\dot{\text{Q}}}$ determination facility of the Oxycon Sigma ${\dot{\text{V}}\text{O}}_{2}$, HR, and ${\dot{\text{Q}}}$ were determined during the final minute of 3 min treadmill running stages at 2.22 m s^−1^ (8 km h^−1^) and 2.50 m s^−1^ (9 km h^−1^) interspersed with a 1-min rest period. Following a further 1-min rest, 35 students (27 boys) satisfactorily completed a third stage running at 2.78 m s^−1^ (10 km h^−1^). ${\dot{\text{Q}}}$ was determined indirectly using the CO_2_ rebreathing technique in accordance with the methodology recommended by Jones (1997). The partial pressure of CO_2_ in arterial blood (PaCO_2_) was estimated from the end tidal CO_2_. The partial pressure of mixed venous CO_2_ (PvCO_2_) was estimated from the rebreathing equilibrium. The downstream correction was applied to the partial pressure of the equilibration CO_2_ to adjust for alveolar to blood partial pressure differences. The CO_2_ content of venous and arterial blood was calculated from PvCO_2_ and PaCO_2_ using the McHardy curve adjusted for the effect of individual differences in haemoglobin (McHardy et al. 1967). The volume of gas in the rebreathing bag was calculated to be 1.5 times the mean of three previous tidal breaths and the bag CO_2_ concentration, which varied from 9 to 13%, was calculated based on the ${\dot{\text{V}}\text{O}}_{2}$ and the end tidal partial pressure of CO_2_. The size of the rebreathing bag was selected individually to accommodate the gas volume, but without being so large as to prevent appropriate CO_2_ equilibrium. Only tests which demonstrated a CO_2_ equilibrium were included in the data (Jones 1997). Individual SVs were calculated by dividing ${\dot{\text{Q}}}$ with HR.

### Data analysis

*Study 1* Data were stored and analysed using SPSS version 25 (IBM, SPSS statistics, Portsmouth, UK). Descriptive data (means and standard deviations) were computed, between-sex differences were explored, and within-sex differences across the exercise stages were examined using ANOVA and paired *t* tests. Significance was set at *p* < 0.05.

*Study 2* To describe relationships between FFM, SV_max_, and peak ${\dot{\text{V}}\text{O}}_{2}$, data were graphed and Pearson product moment correlation coefficients were computed. Data were analysed using multilevel regression modelling (MLWin v3.02, Centre for Multilevel Modeling, University of Bristol, UK). Longitudinal changes in (1) SV_max_ with FFM and age, and, (2) in peak ${\dot{\text{V}}\text{O}}_{2}$ with FFM, SV_max_, and age were analysed using the multiplicative, allometric approach introduced by Nevill et al. (1998) as follows:

1 $$ y = {\text{ FFM}}^{k} \;\cdot{ \exp }\left( {a_{j} + b\;\cdot\;{\text{age}}} \right)\varepsilon_{ij} , $$

where *y* = SV_max_ or peak ${\dot{\text{V}}\text{O}}_{2}$.

Log transformation linearizes the model as in the following equation forming the starting point for analyses:

2 $$ { \log }_{e} \,y = k\;\cdot{ \log }_{e} \;{\text{FFM }} + a_{j} + b\;\cdot{\text{ age }} + { \log }_{e} \left( {\varepsilon_{ij} } \right), $$

where *y* = SV_max_ or peak ${\dot{\text{V}}\text{O}}_{2}$.

All parameters were fixed with the exception of the constant (*a*) which was allowed to vary randomly at level 2 (between individuals) and the multiplicative error ratio (*ε*) which also varied randomly at level 1 (within individuals) as denoted by the subscripts *i* (level 1 variation) and *j* (level 2 variation). Age was centred on the group mean. Other factors associated with the dependent variable were explored either as additional covariates or through the calculation of dummy variables (setting the boys’ constant as baseline and investigating any departure from this for girls) or interaction terms allowing for different relationships between covariates and sex to be examined.

Parameter estimates were considered significant (*p* < 0.05) where their value exceeded 2 × the standard error (SE). Where more than one model was investigated, a comparison of the goodness of fit of the different models was obtained from the change in the deviance statistic (− 2 × log-likelihood) with reference to the number of fitted parameters. The model with the smallest log-likelihood reflects that with the best fit for the same number of fitted parameters. Additional parameters contribute to improved fit from the change in the log-likelihood according to a *χ*² statistic for additional degrees of freedom added.

## Results

*Study 1* Descriptive data are presented in Table 1. Maturity status ranged from 9 boys and 4 girls pre-pubertal (at stage 1 for PH) to 13 boys and 9 girls at stage 2, 9 boys and 11 girls at stage 3, 3 boys and 2 girls at stage 4, and 1 girl at stage 5. There were no significant (*p* > 0.05) sex differences in age, stature, body mass, sum of two skinfolds, FFM, HR at peak ${\dot{\text{V}}\text{O}}_{2}$, or Hb concentration, but boys had a significantly (*p* < 0.01) higher mean peak ${\dot{\text{V}}\text{O}}_{2}$ than girls.

**Table 1:** Study 1: Descriptive data

	Boys (*n* = 34)	Girls (*n* = 27)
Age (years)	12.2 (0.4)	12.1 (0.4)
Stature (m)	1.51 (0.07)	1.52 (0.06)
Mass (kg)	40.2 (6.1)	41.3 (6.1)
Fat-free mass (kg)	33.1 (4.4)	32.6 (3.9)
Haemoglobin (g L^−1^)	135 (7)	133 (7)
Sum of two skinfolds (mm)	19.3 (8.3)	19.9 (5.6)
Peak oxygen uptake (L min^−1^)	2.09 (0.28)	1.83 (0.24)**
Peak heart rate (beats min^−1^)	204 (6)	203 (8)

Values are means (standard deviations). Fat-free mass estimated from youth-specific equations (Slaughter et al. 1988); no significant (*p* > 0.05) sex differences except in **peak oxygen uptake (*p* < 0.01)

Table 2 summarizes exercise responses during consecutive 3 min treadmill running stages at 2.22 and 2.50 m s^−1^. In both sexes, there were significant (*p* < 0.01) increases in ${\dot{\text{V}}\text{O}}_{2}$,  % peak ${\dot{\text{V}}\text{O}}_{2}$ and HR with progressively increasing treadmill speed, but no significant (*p* > 0.05) change in SV (change: boys ~ 2%; girls ~ 3%). A subsample of this group continued with a further exercise bout running at 2.78 m s^−1^. For the 27 boys,  % peak ${\dot{\text{V}}\text{O}}_{2}$ significantly (*p* < 0.01) increased to 78.7 ± 6.4% with a nonsignificant (*p* > 0.05) ~ 3% change in SV to 82.6 ± 11.7 mL. For the 8 girls who also exercised at a treadmill speed of 2.78 m s^−1^,  % peak ${\dot{\text{V}}\text{O}}_{2}$ significantly (*p* < 0.01) increased to 89.4 ± 7.7% with a nonsignificant (*p* > 0.05) ~ 3% change in SV to a mean value of 71.8 ± 7.6 mL recorded.

**Table 2:** Study 1: Exercise data running at treadmill speeds of 2.22 and 2.50 m s^−1^

Treadmill speed	2.22 m s^−1^	2.50 m s^−1^
Boys (*n* = 34)		
Oxygen uptake (L min^−1^)	1.41 (0.23)	1.55 (0.23)**
Percent peak oxygen uptake	67.5 (7.5)	74.3 (7.6)**
Heart rate (beats min^−1^)	163 (14)	173 (13)**
Stroke volume (mL)	81.6 (11.0)	80.1 (11.1)
Girls (*n* = 27)		
Oxygen uptake (L min^−1^)	1.41 (0.23)	1.51 (0.20)**
Percent peak oxygen uptake	77.5 (5.6)	82.9 (7.1)**
Heart rate (beats min^−1^)	176 (11)	183 (11)**
Stroke volume (mL)	76.5 (9.9)	74.6 (9.7)

Values are means (standard deviations). Significant differences between stages **p < 0.01

**Fig. 1:**
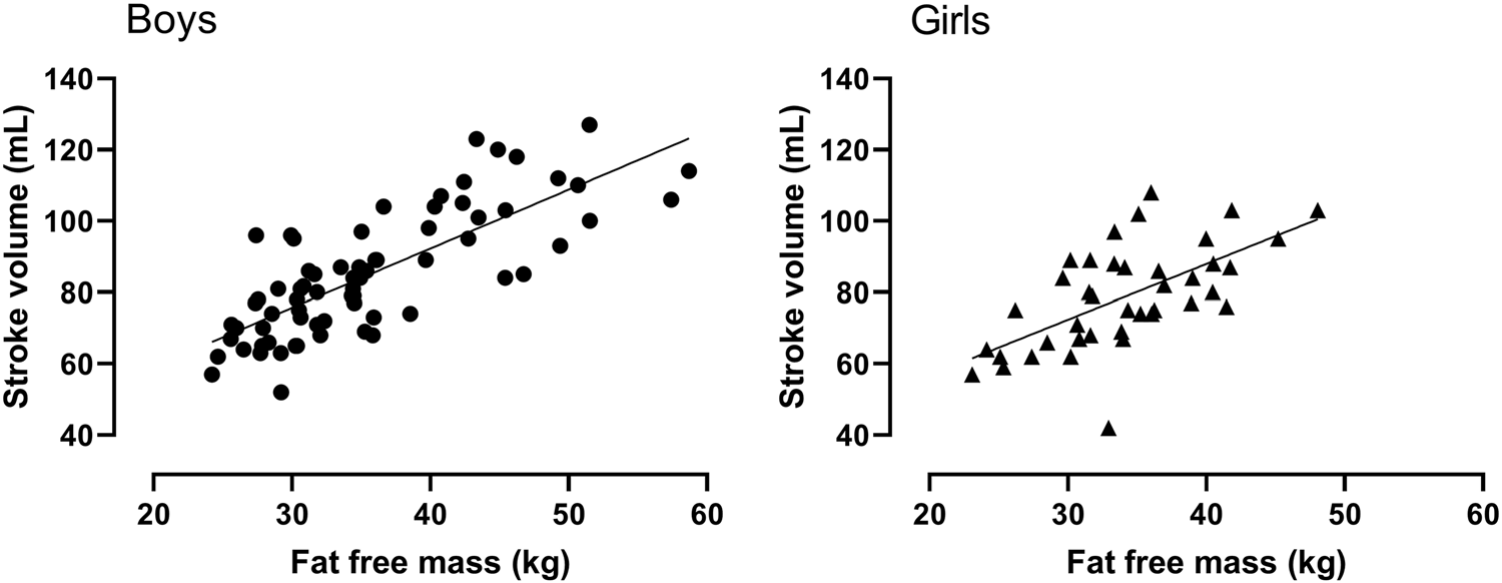
Maximal stroke volume in relation to fat-free mass by sex. Data are from 111 (71 boys) determinations of maximal stroke volume and fat-free mass. Fat-free mass is estimated from youth-specific equations (Slaughter et al. 1988)

**Fig. 2:**
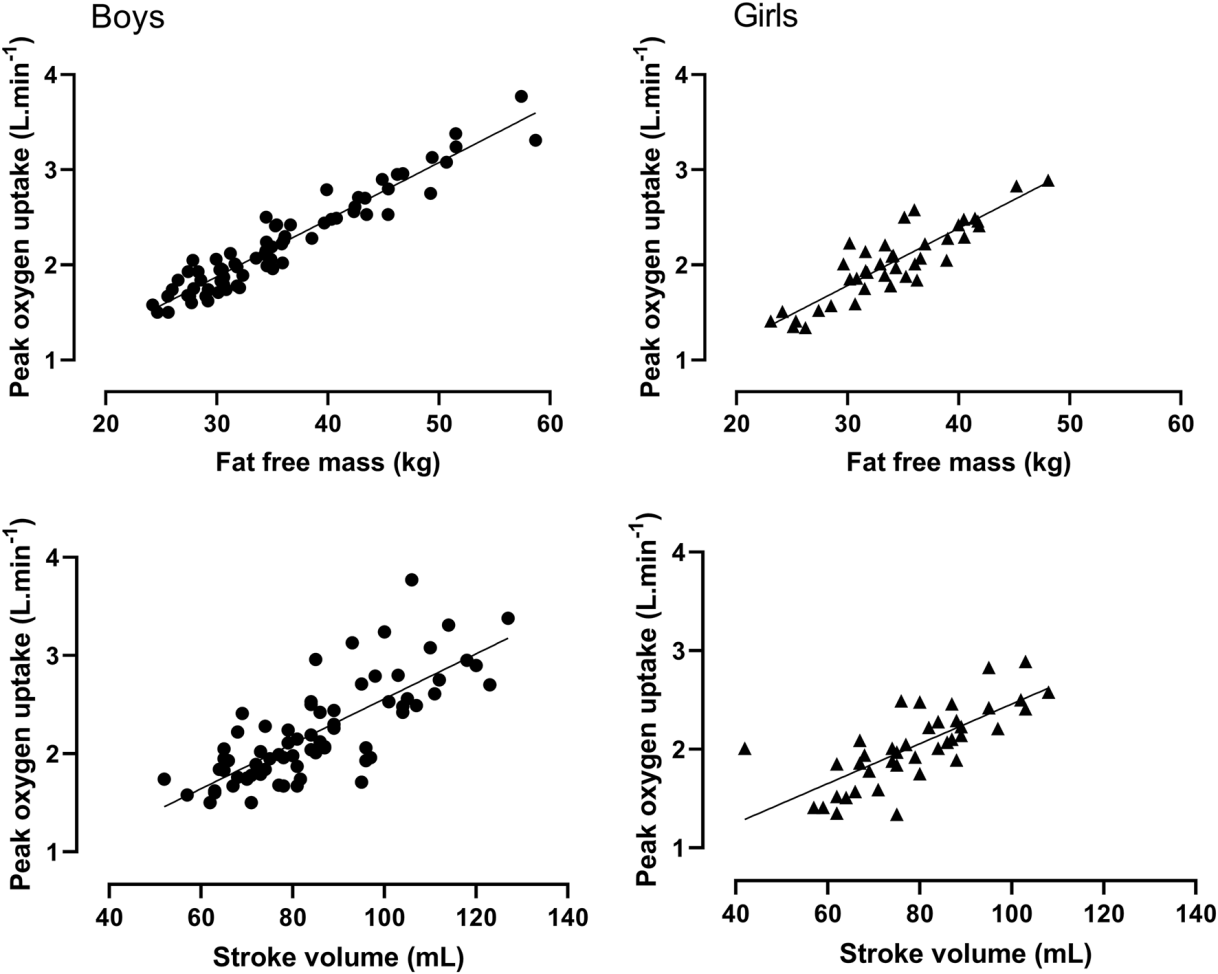
Peak oxygen uptake in relation to maximal stroke volume and fat-free mass by sex. Data are from 111 (71 boys) determinations of peak oxygen uptake, maximal stroke volume, and fat-free mass. Fat-free mass is estimated from youth-specific equations (Slaughter et al. 1988)

*Study 2* Figure 1 illustrates the sex-specific relationships between SV_max_ and FFM. Figure 2 illustrates the sex-specific relationships between peak ${\dot{\text{V}}\text{O}}_{2}$ and FFM, and peak ${\dot{\text{V}}\text{O}}_{2}$ and SV_max_. Significant (*p* < 0.01) correlation coefficients between SV_max_ and FFM were *r* = 0.78 and *r* = 0.62 for boys and girls, respectively. Peak ${\dot{\text{V}}\text{O}}_{2}$ was significantly (*p* < 0.01) correlated with FFM (boys, *r* = 0.96; girls, *r* = 0.89) and with SV_max_ (boys, *r* = 0.78; girls, *r* = 0.74).

In contrast to traditional methods that require a complete longitudinal dataset, both the number of observations per individual and the temporal spacing of the observations can vary within a multilevel analysis. The multilevel analyses presented herein are founded on 111 (71 from boys) determinations of FFM, SV_max_, and peak ${\dot{\text{V}}\text{O}}_{2}$. Table 3 presents multilevel allometric models for SV_max_ with FFM. In model 3.1, log_*e*_ FFM was revealed as a significant (*p* < 0.05) explanatory variable with SV_max_ increasing proportionally to FFM^0.72^. With FFM controlled for, there were no significant sex differences in SV_max_. Models 3.2 and 3.3 describe very similar models for SV_max_ by sex. In boys, the FFM exponent was 0.72 as in the combined sex model and in girls, the FFM exponent was a similar 0.68. Age and maturity status were not significant (*p* > 0.05) factors in any model.

**Table 3:** Study 2: Multilevel allometric models for stroke volume

Response	Log_*e*_ SV		Log_*e*_ SV Boys		Log_*e*_ SV Girls	
	Model 3.1	S.E.	Model 3.2	S.E.	Model 3.3	S.E.
Fixed part						
Constant	1.857	0.241	1.857	0.260	1.962	0.501
Log_*e*_ FFM	0.718	0.068	0.723	0.073	0.679	0.142
Sex	ns		–		–	
Random part						
Level: id						
Var (cons)	0.007	0.003	0.007	0.003	0.007	0.005
Level: time						
Var (cons)	0.011	0.002	0.009	0.002	0.016	0.005
Units: id	51		32		19	
Units: time	111		71		40	
− 2*log-likelihood	− 138.122		− 104.520		− 39.431	

Values are model estimates (standard error); *FFM* fat-free mass estimated from youth-specific equations (Slaughter et al. 1988), *SV* stroke volume, – not entered, *ns* not significant (*p* > 0.05)

Table 4 describes the multilevel allometric models for peak ${\dot{\text{V}}\text{O}}_{2}$. Model 4.1 shows peak ${\dot{\text{V}}\text{O}}_{2}$ to increase in almost direct proportion with FFM, but after adjusting for FFM, girls’ peak ${\dot{\text{V}}\text{O}}_{2}$ was ~ 5% lower than boys’ peak ${\dot{\text{V}}\text{O}}_{2}$. Introducing log_*e*_ SV_max_ as an additional covariate in Model 4.2 yielded a significant (*p* < 0.05) parameter estimate of 0.12 (SE .05) showing an additional effect of SV_max_ on peak ${\dot{\text{V}}\text{O}}_{2}$ and providing a model with a significantly (*p* < 0.05) better statistical fit. However, a significant (*p* < 0.05) ~ 4% sex difference in peak ${\dot{\text{V}}\text{O}}_{2}$ remained. In boys (Model 4.3), once FFM had been controlled for, SV_max_ was not a significant (*p* > 0.05) additional explanatory variable for peak ${\dot{\text{V}}\text{O}}_{2}$. For girls (Model 4.4), both FFM and SV_max_ were significant explanatory variables (*p* < 0.05) with exponents for FFM of 0.93 (SE .09) and SV_max_ of 0.18 (SE 0.08). Age, maturity status, and Hb concentration were not significant (*p* > 0.05) factors in any model.

**Table 4:** Study 2: Multilevel allometric models for peak oxygen uptake

Response	Log_*e*_ peak ${\dot{\text{V}}\text{O}}_{2}$		Log_*e*_ peak ${\dot{\text{V}}\text{O}}_{2}$		Log_*e*_ peak ${\dot{\text{V}}\text{O}}_{2}$ Boys		Log_*e*_ peak ${\dot{\text{V}}\text{O}}_{2}$ Girls	
	Model 4.1	S.E.	Model 4.2	S.E.	Model 4.3	S.E.	Model 4.4	S.E.
Fixed part								
Constant	− 2.706	0.132	− 2.931	0.161	− 2.590	0.140	− 3.347	0.309
Log_*e*_ FFM	0.980	0.037	0.895	0.051	0.947	0.039	0.925	0.093
Log_*e*_ SV	–		0.119	0.050	ns		0.181	0.083
Sex	− 0.046	0.019	− 0.040	0.018	–		–	
Random part								
Level: id								
Var (cons)	0.003	0.001	0.002	0.001	0.002	0.001	0.003	0.002
Level: time								
Var (cons)	0.003	0.001	0.003	0.001	0.002	0.001	0.004	0.001
Units: id	51		51		32		19	
Units: time	111		111		71		40	
− 2*log-likelihood	− 275.520		− 280.698		− 194.700		− 90.828	

Values are model estimates (standard error); *FFM* fat-free mass estimated from youth-specific equations (Slaughter et al. 1988), *SV* stroke volume, ${\dot{\text{V}}\text{O}}_{2}$ oxygen uptake, – not entered, *ns* not significant (*p* > 0.05)

## Discussion

Study 1 empirically confirmed that during late childhood and early adolescence, SV plateaus during progressive treadmill running. Despite an increase in treadmill running speed which induced significant increases in HR, ${\dot{\text{V}}\text{O}}_{2}$, and  % peak ${\dot{\text{V}}\text{O}}_{2}$ in both sexes, SV did not rise but levelled-off with values within ~ 2–3%. The underlying physiology suggests that at the onset of progressive upright exercise, the joint effects of arteriolar vasodilation and the pumping action of the skeletal muscles in the legs mobilize the blood pooled in the legs and increase the venous return to the heart. SV increases from rest by ~ 30–40% before levelling-off and remaining stable with further increases in exercise intensity (Rowland et al. 2000b; Rowland and Unnithan 2013: Tschakovsky et al. 1996). The stability of SV is maintained by the regulation of HR which rises in parallel with the venous return not only to increase ${\dot{\text{Q}}}$ but also to ‘defend’ the optimal SV through to peak ${\dot{\text{V}}\text{O}}_{2}$ (Rowland 2017; Winsley 2007).

The failure of SV to increase further once progressive exercise reaches ~ 50% of peak ${\dot{\text{V}}\text{O}}_{2}$ allows the CO_2_ rebreathing technique to be used in paediatric studies to estimate SVmax. Both the present authors’ observations and the data support the view that children and adolescents find rebreathing CO_2_ to be disagreeable, particularly when exercising near their peak ${\dot{\text{V}}\text{O}}_{2}$ (Driscoll et al. 1989; Washington 1993; Winsley 2007). Notably, increasing the treadmill belt speed to elicit ~ 90% peak ${\dot{\text{V}}\text{O}}_{2}$ reduced participant compliance which resulted in only 8 (~ 30%) girls being willing or able to complete the stage. However, even in these girls, SV did not significantly (*p* > 0.05) change (~ 3%) with an exercise step change from ~ 78 to 89% of peak ${\dot{\text{V}}\text{O}}_{2}$. 27 boys (~ 79%) satisfactorily completed the third stage which was at ~ 79% of peak ${\dot{\text{V}}\text{O}}_{2}$ with a mean SV which was within 1% of that at ~ 68% of peak ${\dot{\text{V}}\text{O}}_{2}$. These data confirm the extant literature (e.g. Rowland 2005) that submaximal SV, in the present case above ~ 67% (boys) and ~ 77% (girls) of peak ${\dot{\text{V}}\text{O}}_{2}$, can be assumed to reflect SV_max_.

The vast majority of studies of paediatric exercise SV have involved cycle ergometry and, with the range of techniques employed to estimate SV_max_, absolute values are not directly comparable across studies (Warburton and Bredin 2017). Within study, sex differences in absolute SV_max_ are, however, consistent and the  % sex differences in SVmax in the present study (~ 6–7%) are in remarkable agreement with those reported elsewhere for children and early adolescents (~ 5–7%) (Rowland et al. 2000a; Turley and Wilmore 1997; Vinet et al. 2003).

SVmax essentially depends on cardiac size and function and although empirical data are sparse, the extant literature suggests that sex differences in SVmax reflect differences in cardiac dimensions (Turley and Wilmore 1997; Vinet et al. 2003). Cardiac dimensions are closely associated with FFM and a potential relationship between skeletal and cardiac muscularity has been suggested (Batterham et al. 1999; George et al. 1991). Vinet et al. (2003) reported that in pre-pubertal children with FFM allometrically controlled for, sex differences in left ventricular dimensions and left ventricular mass were no longer significant (*p* > 0.05). Figure 1 illustrates the relationship between FFM and SVmax supported by significant (*p* < 0.01) correlations of *r* = 0.78 and *r* = 0.62, in boys and girls, respectively.

Model 3.1 (Table 3) shows that SVmax increases in proportion to FFM^0.72^ and with FFM controlled for, there was no significant (*p* > 0.05) sex difference in SVmax. This is in agreement with earlier cross-sectional studies of similar-aged children which also observed sex differences in SV_max_ to be not significant (*p* > 0.05) following allometric normalization with FFM (Rowland et al. 2000a; Vinet et al. 2003). Moreover, these authors reported similar FFM exponents of 0.84 and 0.79, respectively, both of which fall within the 95% confidence limits of the present FFM exponent of 0.72 (0.07). Sex-specific models of SV_max_ were similar with FFM exponents of 0.72 (Model 3.2, boys) and 0.69 (Model 3.3, girls). Once FFM had been controlled for, age and maturity status were not significant (*p* > 0.05) explanatory variables in any model, but this may have been influenced by the limited range of ages and population of stages in maturity status in the present study of 11–13-year-olds. There are no longitudinal studies with which to compare the present data.

The strong influence of FFM on the development of peak ${\dot{\text{V}}\text{O}}_{2}$ across the age range 10–18 years is well documented and has been largely attributed to increases in muscle mass enhancing total muscle ${\dot{\text{V}}\text{O}}_{2}$ and venous return during exercise (Armstrong and Welsman 2019a). In mid- and late-adolescence, a marked increase in FFM is strongly influenced by the timing and tempo of maturation, particularly in boys (Armstrong 2019; Baxter-Jones et al. 2003). Figure 2 illustrates that even with children and early adolescents, there are strong relationships between peak ${\dot{\text{V}}\text{O}}_{2}$ and FFM with similar significant (*p* < 0.01) correlations to those observed by Armstrong and Welsman (2019a) over an age range of 10–18 years in both boys (*r* = 0.96 vs *r* = 0.94) and girls (*r* = 0.89 vs *r* = 0.87). Model 4.1 (Table 4) shows a significant (*p* < 0.05) sex difference in peak ${\dot{\text{V}}\text{O}}_{2}$ of ~ 5% once FFM had been controlled for, with age having no additional significant effect. Whereas, in 10–18-year-olds with FFM controlled for, age had a significant (*p* < 0.05) positive effect on peak ${\dot{\text{V}}\text{O}}_{2}$ supplemented with a significant positive age by sex interaction which resulted in a larger sex difference of ~ 10% (Armstrong and Welsman 2019a).

Figure 2 shows the relationship between peak ${\dot{\text{V}}\text{O}}_{2}$ and SV_max_ in both sexes with significant (*p* < 0.01) correlations in boys and girls of *r* = 0.78 and *r* = 0.74, respectively. The introduction of SV_max_ into Model 4.2 resulted in a significant (*p* < 0.05) positive effect on peak ${\dot{\text{V}}\text{O}}_{2}$, a small reduction to ~ 4% in the sex difference, and a significantly (*p* < 0.05) better statistical fit for the combined sex data. Model 4.4 of the girls’ data shows a similar pattern with SV_max_ having a moderate, but significant (*p* < 0.05) effect on peak ${\dot{\text{V}}\text{O}}_{2}$. On the other hand, in boys, the effect of SV_max_ on peak ${\dot{\text{V}}\text{O}}_{2}$ was small and not significant (*p* > 0.05) once FFM had been controlled for as showed in Model 4.3. Any effects of SV_max_ on boys’ peak ${\dot{\text{V}}\text{O}}_{2}$ appear to be masked by and reflected in the increasing FFM in boys, even during early adolescence.

Fat-free mass is the most powerful morphological influence on the development of peak ${\dot{\text{V}}\text{O}}_{2}$ in both sexes and accounts for much of the increasing sex difference in the development of aerobic fitness during youth. The present data suggest that with FFM controlled for, the sex difference in the peak ${\dot{\text{V}}\text{O}}_{2}$ of children and early adolescents falls from ~ 15 to 5%. SV_max_ makes a statistically significant (*p* < 0.05) contribution to explain the development of peak ${\dot{\text{V}}\text{O}}_{2} ,$ but an unexplained sex difference remains. Why with FFM and SV_max_ controlled for there remains a ~ 4% sex difference in peak ${\dot{\text{V}}\text{O}}_{2}$ is unresolved.

The extant literature provides unequivocal evidence of no sex difference in HRmax (Armstrong and McManus 2017) and strongly suggests no sex differences in maximal A-VO_2_ diff (Rowland 2017), although in this case, there are some conflicting data (Winsley et al. 2009). There were no sex differences in Hb concentration in the present studies, but even when sex differences in Hb are apparent as in late adolescence any effect on sex differences in peak ${\dot{\text{V}}\text{O}}_{2}$ remains to be proven (Armstrong and Welsman 2001). A poorer matching of muscle oxygen delivery to oxygen utilization in 9–10-year-old girls than similar-aged boys has been reported from a study using near infra-red spectroscopy to estimate microcirculatory changes in deoxygenated Hb and myoglobin (McNarry et al. 2015), but this has not been confirmed. Methodological and ethical challenges leave it unclear whether there are meaningful sex differences in the ratio of active muscle mass to FFM, or in muscle structure, or in aerobic enzyme activity, or in muscle fibre types, or in muscle activation (Armstrong et al. 2017; Dotan et al. 2012; Malina et al. 2004). For the physiological mechanisms underlying sex differences in the development of aerobic fitness to be fully elucidated further development and application of non-invasive technology to developmental exercise physiology is required.

### Strengths and limitations of the studies

Limitations to the present study potentially include the methodology of estimating SV at ~ 83% (girls) and ~ 74% (boys) of peak ${\dot{\text{V}}\text{O}}_{2}$ and extrapolating it to represent SVmax. However, the validity of extrapolating SV values from submaximal exercise to SV_max_ is well documented and evidenced using a range of techniques, the data presented herein clearly demonstrate the SV plateau phenomenon (~ 2–3% change) with these participants, and we analysed data as close to peak ${\dot{\text{V}}\text{O}}_{2}$ as reasonable for the young participants, many of whom found CO_2_ rebreathing unpleasant at high exercise intensities. The estimation rather than the direct measurement of FFM can be criticised, but the methodology is well established in paediatric exercise physiology (Slaughter et al. 1988) and ‘direct’ measures of body fat of the same young people have recently been shown to vary widely across laboratory techniques (Ferri-Morales et al. 2018). Moreover, FFM includes tissues not involved in exercise and ideally active muscle mass would be directly determined on each test occasion. This is not currently feasible in paediatric exercise studies involving over 120 multiple assessments. Another limitation is that although the study was longitudinal, it only covered a 2 year period. On the other hand, a unique strength of the study lies in the provision for the first time of longitudinal insights into the development of SV_max_ and its influence on peak ${\dot{\text{V}}\text{O}}_{2}$ in healthy, untrained children and early adolescents. A major strength lies in the adoption of a multiplicative allometric modelling approach which allowed variables to be partitioned concurrently within an allometric framework to provide sensitive interpretations of the development of SV_max_ and peak ${\dot{\text{V}}\text{O}}_{2}$.

## Conclusions

Study 1 confirms that during progressive treadmill running, SV levels-off and reaches its maximum at submaximal intensities. Although there are no comparable longitudinal studies, Study 2 data are in accordance with cross-sectional analyses of children and early adolescents and demonstrate that there are no significant sex differences in SV_max_ once FFM has been appropriately controlled for. Multilevel allometric models demonstrate that FFM exerts a powerful influence on the development of peak ${\dot{\text{V}}\text{O}}_{2}$ but even with FFM controlled for, there remains a ~ 5% sex difference. SV_max_ makes a modest additional contribution to explaining the development of peak ${\dot{\text{V}}\text{O}}_{2} ,$ but a residual ~ 4% sex difference in peak ${\dot{\text{V}}\text{O}}_{2}$ remains unexplained.

## Abbreviations

A-VO_2_ diff: Arteriovenous oxygen difference
BSA: Body surface area
CO_2_: Carbon dioxide
$$ {\dot{\text{Q}}} $$: Cardiac output
FFM: Fat-free mass
Hb: Haemoglobin
HR: Heart rate
Max: Maximal
$$ {\dot{\text{V}}\text{O}}_{2} $$: Oxygen uptake
PaCO_2_: Partial pressure of CO_2_ in arterial blood
PvCO_2_: Partial pressure of mixed venous CO_2_
PH: Pubic hair
SV: Stroke volume
